# Large Doses of Pills for Children

**Published:** 1854-05

**Authors:** 


Large Doses of Pills for Children.—A child in this city, three years old, was left alone by its parents last week, for a short time, when it improved the opportunity of imitating their mode of medication, by resorting to the pill-box. It appears that a box of socalled "Indian Vegetable Pills" had been left within its reach, and the child actually took *eleven* of them! But, strange to say, no harm followed this extraordinary large dose, save the fright it caused his distressed parents. It did *physic* the little one pretty thoroughly, and *we are told* that the child's health has been improved by the accidental circumstance!

Another similar case recently occurred under our observation. A child, who had a peculiar liking for pills, during the absence of its nurse went to the medicine chest and took from it a small vial containing *homœopathic* globules, said to be "the active properties of *dulcamara*," and of which it was considered that "*three* was a large dose for an adult." The little urchin actually ate two-thirds of them, say about *one hundred!* We were consulted by the parents immediately after the *accident* was made known to them; but on learning the facts we assured them they need have no fear of any bad results, as such infinitesimals could not possibly do the child an injury. Since then, we understand the parents have abandoned the use of such very "*dangerous medicines*," and now employ a regular practitioner.—*Boston Medical and Surgical Journal.*

